# Metapopulation structure for perpetuation of *Francisella tularensis tularensis*

**DOI:** 10.1186/1471-2180-9-147

**Published:** 2009-07-23

**Authors:** Heidi K Goethert, Benjamin Saviet, Sam R Telford

**Affiliations:** 1 Division of Infectious Diseases, Tufts Cummings School of Veterinary Medicine, 200 Westboro Rd, North Grafton, MA 01536, USA

## Abstract

**Background:**

Outbreaks of Type A tularemia due to *Francisella tularensis tularensis *are typically sporadic and unstable, greatly hindering identification of the determinants of perpetuation and human risk. Martha's Vineyard, Massachusetts has experienced an outbreak of Type A tularemia which has persisted for 9 years. This unique situation has allowed us to conduct long-term eco-epidemiologic studies there. Our hypothesis is that the agent of Type A tularemia is perpetuated as a metapopulation, with many small isolated natural foci of transmission. During times of increased transmission, the foci would merge and a larger scale epizootic would occur, with greater likelihood that humans become exposed.

**Methods:**

We sampled questing dog ticks from two natural foci on the island and tested them for tularemia DNA. We determined whether the force of transmission differed between the two foci. In addition, we examined the population structure of *F. tularensis *from ticks by variable number tandem repeat (VNTR) analysis, which allowed estimates of diversity, linkage disequilibrium, and eBURST analysis.

**Results:**

The prevalence of tularemia DNA in ticks from our two field sites was markedly different: one site was stable over the course of the study yielding as many as 5.6% positive ticks. In contrast, infected ticks from the comparison site markedly increased in prevalence, from 0.4% in 2003 to 3.9% in 2006. Using 4 VNTR loci, we documented 75 different haplotypes (diversity = 0.91). eBURST analysis indicates that the stable site was essentially clonal, but the comparison site contained multiple unrelated lineages. The general bacterial population is evolving clonally (multilocus disequilibrium) and the bacteria in the two sites are reproductively isolated.

**Conclusion:**

Even within an isolated island, tularemia natural foci that are no more than 15 km apart are uniquely segregated. One of our sites has stable transmission and the other is emergent. The population structure at the stable site is that of a clonal complex of circulating bacteria, whereas the emerging focus is likely to be derived from multiple founders. We conclude that the agent of tularemia may perpetuate in small stable natural foci and that new foci emerge as a result of spillover from such stable sites.

## Background

The perpetuation of *Francisella tularensis tularensis*, the agent of Type A tularemia, has been argued to depend upon cottontail rabbits [1-3], and until relatively recently, most human cases have indeed been associated with hunting or processing these animals [4]. Cases now appear to mainly be due to tick exposure. [5] Although many different kinds of hematophagous arthropods are competent vectors in the laboratory, only dog ticks (*Dermacentor andersoni *and *D. variabilis*; [6,7], Lone Star ticks (*Amblyomma americanum*; [8] and tabanid flies (*Chrysops spp*.; [9] are thought to be zoonotic vectors in the United States. The mode of perpetuation seems to involve a combination of horizontal (infection of various vertebrates, which in turn infect new ticks or flies) and vertical (inheritance of infection by tick progeny) transmission [10], but the relative importance of either mechanism remains to be measured. Identifying sites of transmission largely depends on epizootic activity, particularly outbreaks of human disease. Human Type A outbreaks manifest as a small number of cases, with reports ending quickly as the epizootic rapidly disappears [5], probably due to the mortality of the putative rodent reservoirs. This sporadic nature of Type A epidemiology has greatly hindered identifying the determinants of perpetuation and human risk.

The island of Martha's Vineyard, Massachusetts is unique in the ecology of Type A tularemia in that it is the site of a sustained outbreak of the disease. Nearly 90 human cases have been identified there since 2000 (Massachusetts Department of Public Health, personal communication). Although ulceroglandular disease is the most commonly reported form of tularemia in the U.S., the majority of the 90 cases reported during 2000–2008 on Martha's Vineyard have presented with the pneumonic form of the disease [11]. A large proportion of the case-patients worked as landscapers: a case control study implicated lawn mowing and brush cutting as high risk activities, but the nature of the fomites remains undescribed [12]. In addition to the distinctive presentation of disease, the Martha's Vineyard tularemia outbreak is unique in its longevity in that cases have occurred for 9 consecutive years. This prolonged epizootic may represent a new level of transmission on the island. In our longitudinal studies of tularemia epidemiology there, we identified dog ticks, *Dermacentor variabilis*, as fundamental to the perpetuation of *F. tularensis tularensis*. Dog ticks appear to be the mode of exposure for the ulceroglandular cases that have been identified there. The main hosts for adult dog ticks (skunks and raccoons) are commonly seropositive whereas no other animal appears to be commonly exposed [13]. Prevalence of *F. tularensis *DNA in dog ticks collected from sites throughout the island and over the course of the outbreak ranges from < 1% to 5%. And, the start of the outbreak in 2000 was associated with an island wide increase in dog ticks [11]. Thus, by focusing on the ecology of dog ticks and in particular, by using them as sampling devices, we may better understand the perpetuation of Type A tularemia.

Molecular epidemiological methods have greatly enhanced our capacity to analyze microbial population structure. The description of variable number tandem repeat (VNTR) loci for *F. tularensis *now allows the discrimination of individual strains. Using VNTR analyses (also known as multilocus variable number tandem repeat analysis, MLVA), we demonstrated previously that the diversity of *F. tularensis tularensis *in dog ticks from Martha's Vineyard is as great as that measured for all existing *F. tularensis *isolates from across North America [14,15]. This suggests that the current outbreak of tularemia is not due to a recent introduction event, but that the agent has been endemic on Martha's Vineyard since its likely introduction in the 1930s. Tularemia has long been classified as an infection of natural focality/nidality. The agents for such infections survive for extended durations, decades or longer, in discrete sites ("natural foci") characterized by specific faunal, floral, and physical associations. [16] We have subsequently confirmed, by the use of GIS mapping and VNTR analysis, the natural nidality of *F. tularensis tularensis *on Martha's Vineyard. [17] Ultimately, we seek to better understand the factors that serve as the basis for epizootics as opposed to cryptic maintenance within natural foci. Our hypothesis is rooted in metapopulation ecology [18,19]: that *F. tularensis tularensis *exists in multiple small, isolated natural foci, in which genetic drift increases diversity until some adaptive equilibrium is achieved. When local conditions change, such as increased density of hosts for subadult dog ticks, "valleys" between such adaptive peaks are traversed and certain strains escape to mix into other "peaks" or establish new ones. Natural selection then operates to homogenize the genetic structure across the metapopulation of natural foci. As a first step in exploring this hypothesis, we examined the population structure of two different sites that are separated by 15 km on the island, a natural focus that has long-term stable transmission and a focus that is newly emerging. In particular, we sought to determine whether the force of transmission between the two sites differed, and using VNTR analysis of *F. tularensis *DNA from host seeking dog ticks, we sought evidence for their genetic isolation.

## Methods

### Tick collection

**Figure 1:**
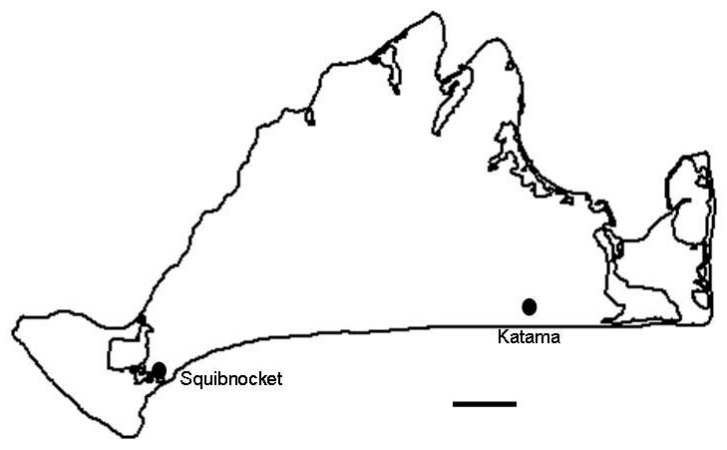
**Collection sites on Martha's Vineyard**.

Collections were conducted from 2003–2007 monthly from April to August. Questing *D. variabilis *were obtained by flagging the vegetation. Additional ticks were obtained by removing them from skunks and raccoons (< 6% of the ticks included in the study) as previously described. [13] Sampling was done from two field sites on opposite sides of the island, near Squibnocket and Katama (see Figure 1). The Squibnocket site is what we believe to comprise a longstanding elementary focus. In contrast, Katama is a site where *D. variabilis *is exceedingly dense but where *F. tularensis tularensis *appears to be rare. Both sites are similar in physiography, with coastal grassland and beach scrub proximal to large brackish water ponds. Both are undeveloped areas of glacial outwash plains with scrubby barrier beach habitat, although the Katama site experiences intensive seasonal use by people for beach access.

### PCR

A drop of hemolymph was obtained from each tick by cutting the front foreleg. This was placed in a tube containing 50 ul PBS. Ticks were processed in pools of 6. Ticks were held at 15°C in individual tubes during screening. The hemolymph pools were boiled for 15 minutes and then used directly as template for PCR. Pools were screened for *F. tularensis tularensis *with a nested PCR reaction targeting the fopA gene as described previously. [14] These primers were chosen for their proven sensitivity and specificity for *F. tularensis tularensis*, as virtually all *D. variabilis *on Martha's Vineyard have been shown to be infected with *Francisella *endosymbionts. [20] Negative controls were included with every PCR. Ticks from PCR-positive pools were reprocessed individually. A drop of hemolymph was placed in a tube with 25 ul PBS, boiled and then amplified by PCR. PCR was not conducted on individual ticks in years in which the prevalence of PCR positive pools was 1% or less. It was deemed unlikely that multiple ticks within a pool would yield positive results. Therefore, the estimates and confidence intervals for the prevalence in low years are maximum likelihood estimates calculated using the Pooled Infection Rate V2.0 Excel Add-In http://www.cdc.gov/ncidod/dvbid/westnile/software.htm. Prevalence estimates and confidence intervals from individual tick data were calculated using the web-based calculators at Statpages.net http://statpages.org/confint.html. Test for trend was done using PEPI v4.0.

### Multiple loci variable number tandem repeat analysis (MLVA)

Amplification of VNTR loci was done directly from the hemolymph lysates as described previously [14,15]. Briefly, PCR was done using a high fidelity Taq polymerase (Picomaxx, Stratagene) and a fluorescently labeled primer (either FAM or HEX). The size of the amplicons was then determined using a capillary sequencer (University of Maine Sequencing Facility, Orono, ME) using GeneMapper software (Applied Biosystems). Each sample contained a DNA ladder for accurate size determination, ABI500 (Applied Biosystems) or MapMarker1000 (BioVentures, Inc.) depending on the expected size of the fragment. These VNTR loci were shown previously not to amplify the *Francisella*-like endosymbionts found in our ticks [12] by specifically using them to test whole tick extracts that were determined to be negative for *F. tularensis *by PCR targeting the fopA gene. Samples with known sizes, such as those derived from the well characterized Live Vaccine Strain (LVS, *F. tularensis holarctica*) or Schu S4 (*F. tularensis tularensis*), were included to assess the consistency from run to run. Peak data were analyzed manually using STRand (Veterinary Genetics Lab, University of California) or Peak Scanner Software v1.0 (Applied Biosystems).

Our previous work demonstrated that locus Ft-M3 (previously called SSTR9) and Ft-M10 (previously SSTR16) are diverse and informative at our field site [14]. These 2 loci were therefore amplified from all samples. Since that work was done, 25 VNTR loci have been developed for the characterization of *Francisella *isolates from a global scale [21]. Due to the limited amount of available template from tick hemolymph, we selected for our analysis loci thought to be potentially informative (demonstrated heterogeneity), based on the published diversity estimates for *Francisella tularensis tularensis *type AI [21]. Selected samples representative of the known diversity on Martha's Vineyard were chosen to test new loci. If no variation was detected for a particular locus, it was not pursued further. The VNTR loci used in this study are: Ft-M3 (SSTR9), Ft-M10 (SSTR16), Ft-M2, Ft-M6, Ft-M8, and Ft-M9. All were amplified as previously described. [14,15] The Ft-M2 locus had a high rate of amplification failures compared to the other loci tested. 16% of the FopA positive ticks successfully amplified all other loci but not Ft-M2. Ticks that had data from the other 3 loci were included in the diversity estimates that did not include the Ft-M2 locus. However, they were necessarily excluded in analyses that include the Ft-M2 locus. Both analyses are presented here.

The number of repeat units for each locus was determined by comparing the obtained amplicon size with one that has a known number of repeats, such as Schu. VNTR haplotypes were then expressed as the number of repeat units. Some samples contained multiple peaks that were not likely to be stutter peaks. These samples were scored as multiple alleles if the amplitude of the smaller peak was > 25% of the larger. These samples were then counted twice, once for each allele, in the MLVA. Simpson's Index of Diversity was calculated as described previously. [22]

### eBurst Analysis

The data from each field site was analyzed using eBURST http://eburst.mlst.net/. [23] eBURST displays the relationships between closely related samples from a bacterial population (e.g. [24,25] It uses an algorithm to identify the founder of the population, by identifying the VNTR type that differs from more of the others by only one locus (single locus variants). It then predicts a likely evolutionary path by connecting VNTR types that differ by one locus and displays them as radial links to the founder. The confidence level for the founder is then calculated using 1000 bootstrap replicates.

### Population Structure Analysis

The population structure of *F. tularensis tularensis *on Martha's Vineyard was analyzed using Multilocus http://www.agapow.net/software/multilocus/. [26] Samples from Squibnocket and Katama were tested to determine whether there was linkage disequilibrium among the loci by calculating the index of association. Randomized datasets (100) that shuffle the alleles among individuals, independently for each locus, were compared to the observed data to calculate statistical significance (set *a priori *at P < 0.05). Evidence for differentiation between the two populations was found using Weir's formulation of Wright's F_st _for haploids. Randomizations were used to calculate significance for this statistic also. In this case the observed data was compared to datasets of the individuals randomized across populations.

### Animal care and use

Trapping of animals was conducted under an approved Institutional Animal Care and Use Committee protocol (G2009-18) at Tufts University, as well as a scientific collecting permit from the Massachusetts Division of Fish and Wildlife.

## Results

**Figure 2:**
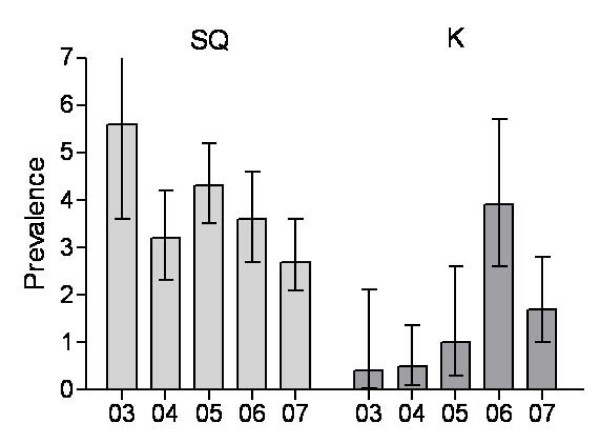
**Estimates of the prevalence (percent infected with 95% confidence intervals) of *F. t. tularensis *in questing *D. variabilis *2003–2007 from Squibnocket and Katama**.

To determine whether two sites on the same island may represent differing durations of enzootic activity, ticks were collected for 5 years (2003–2007) from sites on opposite ends of Martha's Vineyard, near Squibnocket and Katama (Figure 1). *F. tularensis tularensis *was intensely maintained throughout the course of the study near Squibnocket; prevalence estimates ranged from 2.7 to 5.6% (Figure 2) with no significant changes between years. In contrast, ticks testing positive for *F. tularensis tularensis *from Katama were relatively rare at the beginning of the study. In 2003 and 2004, the prevalence estimate is 0.5% (Figure 2). Over the course of the study, the number of PCR positive ticks collected from this area significantly increased (P = 0.017 test for trend), reaching levels that are equivalent (inasmuch as the 95% confidence intervals overlap) to those detected on Squibnocket in 2006 and 2007. Thus, one site may be classified as newly emergent (Katama) and the other longstanding.

Using MLVA, we derived a preliminary description of the population structure of *F. tularensis tularensis *within the two sites. Over the course of the study, we obtained 340 ticks that tested positive for *F. tularensis tularensis *by PCR using a nested reaction to the FopA gene. MLVA was then done directly from the tick hemolymph extracts. Ft-M2, Ft-M6, Ft-M8 and Ft-M9 were all tested on a subset of ticks from multiple years. Ft-M6 and Ft-M8 yielded identical results from all ticks tested, and it was not deemed worthwhile to pursue these loci further. All tick extracts therefore were amplified for Ft-M3, Ft-M10, Ft-M9 and Ft-M2. Only those samples, 315 (93%), that readily amplified all (with the exception of Ft-M2) VNTR loci were included in the study. Ft-M2 was not a robust set of primers; 16% of ticks that amplified with the other 3 loci failed to amplify with Ft-M2. The resulting estimate for genetic diversity on Martha's Vineyard was surprisingly large, consistent with our previously reported results. [14] Using only 4 loci, 75 different haplotypes (Table 1) were identified yielding an overall Simpson's Index of Diversity (D) of 0.91 (Table 2). The diversity at each individual locus varied greatly. Ft-M9 had the least amount of diversity (D = 0.05), with only 2 alleles identified, while Ft-M2 had greater diversity (D = 0.81), with 22 alleles identified. Inclusion of the Ft-M2 locus greatly increased the diversity found in our sites (without Ft-M2 D = 0.67, with Ft-M2 D = 0.91); the number of haplotypes rose from 28 to 75. Although the number of positive ticks was significantly less for Katama than Squibnocket, the calculated diversity was greater for every locus analyzed. This is particularly evident with the Ft-M10 locus; SQ D = 0.32, K D = 0.77 (Table 1). One VNTR haplotype 10 7 4 30 predominated on Squibnocket. Almost a third (30.2%) of *F. tularensis tularensis *detected on this site has this single haplotype. The adaptive equilibria of these two natural foci were distinct, as measured by bacterial genetic diversity.

**Table 1:** VNTR haplotypes found on Martha's Vineyard 2003–2007.

Squibnocket					Katama				
M3	M10	M9	M2	total	M3	M10	M9	M2	total
9	7	4	29–37	17	20	11	4	21–33	9
10	7	4	17–35	183	16	15	4	18–20	5
11	7	4	17–38	29	20	9	4	23–30	9
10	4	4	30–31	14	20	12	4	32–33	3
10	8	4	15–32	4	19	11	4	32	1
10	9	4	17	1	19	11	5	30	2
8	10	4	27	2	18	10	5	30–31	2
8	9	4	25–27	9	18	9	4	24	1
11	9	4	20–35	3	16	14	4	19–23	4
11	8	4	30–38	7	16	16	4	19	1
9	4	4	30	1	19	17	4	18	1
10	21	5	27	1	19	9	4	31	1
9	13	5	32–33	2					
11	8	5	35	1					
13	7	4	-	1					
8	7	4	17	1					

The population structure of *F. tularensis tularensis *within *D. variabilis*, as determined by MLVA, is consistent with a population that is evolving clonally. The population showed significant multilocus disequilibrium, (IA = 0.66, P = < 0.01). Furthermore, our data are consistent with the assertion that *F. tularensis tularensis *from Squibnocket and Katama are reproductively isolated (test for population differentiation theta = 0.37, P < 0.01). The VNTR haplotypes from Squibnocket were unique from those originating in Katama (Table 1). Although the Ft-M2 and Ft-M9 loci had alleles common to both sites, the Ft-M3 alleles were completely unique and non-overlapping. We conclude that there has been little or no gene flow between the two natural foci.

EBURST analysis of the *Francisella tularensis tularensis *populations from each field site resulted in very different patterns. VNTR haplotypes from Squibnocket yielded a star diagram. Virtually all the samples could be linked to the putative founder, 10 7 30 (Figure 2A) and are likely to be direct descendents. Of 276 samples, only 12 were outliers that could not be traced back to the founder via single locus variants. EBURST calculated an 89% confidence in 10 7 30 as the founder. This is supported by the fact that this is the single most prevalent haplotype. In contrast, the depicted pattern of Katama is one with multiple groups and a great number of outliers that could not be connected to any others by single locus variants (Figure 2). Three major groups were detected along with one doublet and 4 single outliers. Thus, the emergent Katama natural focus is derived from multiple founders and appears to not have had time for any effect of stabilizing selection.

## Discussion

Describing the mode of perpetuation of *F. tularensis tularensis *in nature has heretofore been elusive because transmission appears to be unstable, unlike that of Type B (*F. tularensis holarctica*) which may persist in water [16,27,28]. Like rabies or plague, tularemia epizootics tend to be short-lived within discrete sites because mammal hosts die very quickly and leave few susceptibles. Nonimmune hosts are a requirement for maintaining a pathogen for which horizontal transmission is the main mode of perpetuation. The sustained nature of the outbreak on Martha's Vineyard provides a unique opportunity to longitudinally analyze *F. tularensis tularensis *in nature. Over the course of 5 years, enough host seeking ticks with *F. tularensis *DNA were collected so that a preliminary analysis of the agent's population structure could be performed. In our site near Squibnocket, we consistently detected a great prevalence of infection throughout the study, demonstrating that we have detected an elementary focus. [17] In contrast, very few ticks from Katama contained *F. tularensis *DNA during the first years of our study, but this site demonstrated a marked increase in prevalence suggestive of an emerging site of transmission.

Although 25 VNTR loci have been previously described for Ft [21], we chose to utilize only 4 in this study. An important factor for this decision is that tick hemolymph samples were limited; the original reports of VNTR analyses worked with an unlimited supply of in vitro cultivated organisms [15,21,29]. However, the use of a small number of informative loci is justifiable because we are studying a small population of microbes that are all closely related. The loci that were chosen were among the fastest evolving and have been shown to be among the most variable of the extant strains of *F. tularensis tularensis*. The 4 loci we used provided great capacity to discriminate presumably clonal bacterial lineages circulating in our study sites. Furthermore, they provide ample signal to enable us to get a first glimpse of the structure of *F. tularensis tularensis *populations there. Unlike most other studies that utilize MLVA, ours focuses on microbial populations present in a very small geographical area and requires the fine resolution of hyper-variable markers. Many of the other VNTR loci described for *F. tularensis tularensis *are more slowly evolving, likely to be invariant within a small geographic area, and therefore uninformative in the context of our study. Indeed, we demonstrated that this was the case for Ft-M6 and Ft-M8.

*F. tularensis tularensis *has been commonly characterized as an infection of natural focality, maintained in cryptic microfoci of transmission [17,30-34]. Such foci may remain largely isolated between epizootics and therefore, genetic drift would tend to foster unique genetic structure within each. Under a model based in metapopulation ecology, such small isolated foci diverge and attain adaptive equilibria associated with the local biocenosis. Epizootic conditions cause such foci to coalesce and become more homogenously distributed via the development and emergence of new foci. Once epizootic conditions wane, transmission ceases in such overflow areas and the original natural foci remain. We found evidence that (1) two foci are genetically isolated; and (2) the newly emergent focus comprised numerous unrelated haplotypes.

As a corollary, we would expect that *F. tularensis tularensis *sampled from a single longterm microfocus would be less diverse due to stabilizing selection. In fact, *F. tularensis *from Squibnocket has by all measures (Table 2) less diversity than that from Katama, despite the fact that approximately 5 times more samples were typed. This is primarily due to the large predominance of a single haplotype, 10 7 4 30. In contrast, *F. tularensis *from Katama does not have a single dominant haplotype but a few equally frequent haplotypes. Taken together, these observations suggest that our metapopulation model for *F. tularensis *perpetuation is empirically based.

**Table 2:** Diversity of VNTR loci over the course of the study: 2003–2007 for Squibnocket and 2004–2007 for Katama.

	Squibnocket			Katama			Together		
Loci	D	No. alleles	No. repeats	D	No. alleles	No. repeats	D	No. alleles	No. repeats
Ft-M3 (SSTR9)	0.45	5	8–13	0.56	4	16–20	0.58	9	8–20
Ft-M10 (SSTR16)	0.32	7	4–21	0.77	8	9–16	0.48	13	4–21
Ft-M9	0.04	2	4–5	0.09	2	4–5	0.05	2	4–5
Ft-M2	0.78	20	15–38	0.91	11	18–33	0.81	22	15–38
Ft-M3, M10, M9	0.56	16	na	0.83	12	na	0.67	28	na
All	0.88	52	na	0.96	23	na	0.91	75	na

(Ft-M6 and Ft-M8 were omitted because they are invariant)

**Figure 3:**
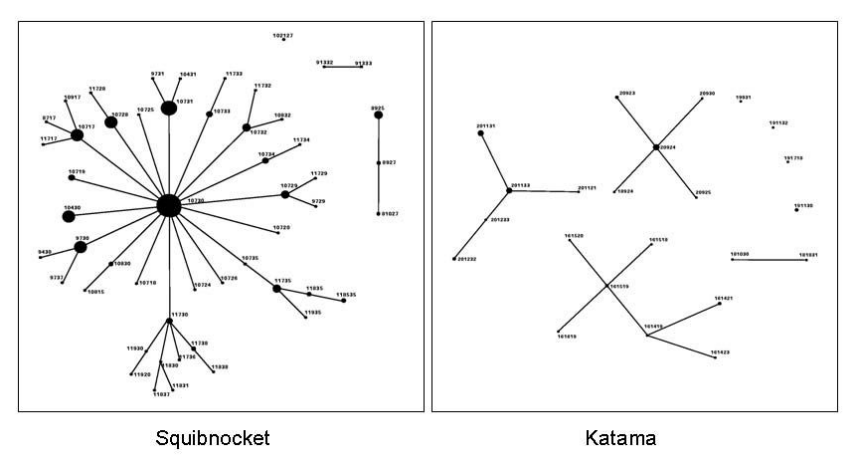
**eBURST analysis of *F. t. tularensis *VNTR haplotypes from questing *D. variabilis *collected comparing Squibnocket, an established site of transmission, to Katama, a newly emerging site**.

Analysis of the population structure of the samples from Squibnocket using eBURST yielded a star diagram indicative of a clonal complex of circulating bacteria (Figure 3). The vast majority of the population of *F. tularensis *from Squibnocket is likely to be related to each other. Greater than 95% of the sampled population of haplotypes can be connected by single locus variants. The putative founder, 10 7 30, is also the dominant haplotype. This structure is consistent with the hypothesis that our site on Squibnocket is indeed a single focus of transmission. Analysis of multilocus linkage disequilibrium in our study was consistent with a clonal population. New alleles are generated primarily through slip-strand mispairing of the repeat regions during replication. Therefore, the rate of generation of new alleles is directly related to the rate of replication and the number of generations. Long-term foci maintaining high levels of transmission would then be expected to generate new haplotypes constantly. Furthermore, the majority of the new haplotypes are expected to be progeny of the ones currently circulating.

Recently, we conducted a study in which we mapped, using a hand-held global positioning system (GPS), the distribution of ticks testing positive for *F. tularensis *on our Squibnocket field site. We found that the vast majority of PCR positive ticks all derived from the same small area within the field site and that they significantly clustered. [17] Furthermore, we noted that ticks collected from the cluster were 3.4 times more likely to contain an uncommon haplotype (i.e., not 10 7). We concluded that there was one focus of transmission in our site on Squibnocket and that this area was the source of genetic diversity there.

In contrast to the star diagram from Squibnocket, the eBURST analysis of *F. tularensis *from Katama depicts 3 groups of haplotypes as well as a doublet and 4 singles (Figure 2). This type of diagram is what would be expected from an area with newly emerging transmission due to multiple recent introduction events. It may be that the diverse and unrelated haplotypes are the result of spillover from multiple foci. Furthermore, it is likely that the sources of the introductions were from nearby areas of Martha's Vineyard. Although we do not have recent data, our previous work demonstrates that other sites in the eastern portion of the island had haplotypes that are close to (i.e., 1 or 2 repeats different) those found at Katama in this study and very different from those found at sites farther away, such as those from Squibnocket [14]. This observation would appear to continue to be valid inasmuch as the current haplotypes from Squibnocket are distinct from that collected in Katama and show evidence of population differentiation. Interestingly, Katama haplotypes detected early in our study (2003 and 2004) do not appear to have amplified over the years and are all singlet outliers, suggesting that not all introduced variants will perpetuate. The haplotypes comprising the 3 groups were all detected later, 2005–2007, consistent with increased enzootic transmission at Katama.

There are several ways in which *F. tularensis *could become introduced into Katama. The Katama field site is near a public beach and a popular surf-fishing site. Skunks and raccoons, hosts for the adult stage of *D. variabilis*, frequent the beach to forage refuse left by beach-goers, to feed on bird eggs laid on the sand, and to steal fish and their entrails from fishermen. Those animals visiting from nearby areas could drop infected replete female *D. variabilis*, which might give rise to infected clusters of larvae. Although the contribution of transovarial transmission to the perpetuation of *F. tularensis *is undetermined, laboratory experiments demonstrate that it may occur [35] but consistent results have not been obtained. (see [6]). In addition, nymphal *Haemaphysalis leporipalustris *or *Ixodes dentatus*, infected as larvae feeding on cottontail rabbits, may be dropped by the area-wide movement of passerine birds, thereby introducing *F. tularensis *into new foci.

Previous studies using tandem-repeat markers have focused on the diversity of strains isolated world-wide or on typing a few strains from small isolated outbreaks. Even when all 25 VNTR loci [2] were tested, these studies showed very little diversity among epidemiologically-related strains. However, these studies all focused on Type B tularemia, the ecology and epidemiology of which is very different than that of Type A. The mutation rate of tandem-repeat markers has been determined in vitro for *E. coli *and plague by serial plating of bacterial colonies. These studies suggest that both bacterial species have similar rates of mutation (i.e., calculated slope of the regression line of repeat copy number versus mutation rate), leading to a general model governing the expected mutation rate of tandem repeats based solely on the number of repeats. [36,37]. However, this model is based solely on in vitro results, and it is not known whether it is applicable for natural transmission cycles. The diversity that we detect for Type A on Martha's Vineyard is very different compared to that reported for epidemiologically-related Type B strains. [15,29] It may be that the mutation rates for the VNTR loci differ for the two *Francisella *subspecies. Alternatively, the differences may be explained by sampling bias (strains isolated in vitro from cases with disease compared to amplicons directly obtained from ticks without isolation). Studies comparing the mutation rates of all the subspecies of *Francisella tularensis*, including Type AI and AII, would appear to be needed to resolve these issues.

When we initiated this long-term study, we were uncertain whether such uncharacterized hypermutating markers would remain stable enough to comprise useful genetic markers years later. Although we infer that a large amount of mutation has occurred through the years in our site, demonstrated by the great diversity of haplotypes, it is clear that clonal lineages are readily identifiable. Only locus Ft-M2 showed excessive diversity and had repeat types clearly indicative of homoplasy. Of particular interest is that identifiable lineages remained stable for years. We first detected our major Squibnocket haplotype (10 7) in 2002 [14]: this was the most prevalent haplotype there in 2002 and still is. Furthermore, analysis of isolates from the human fatality in 2000 yielded a haplotype (11 7) that we have detected on Squibnocket from 2003 to 2007, evidence that this haplotype has been circulating on Martha's Vineyard for at least 8 years[3] Accordingly, although we do not fully understand how stable VNTR markers are for *F. tularensis tularensis*, empirical evidence from our study site suggests that at least some are useful over years of natural transmission.

The results we obtained from the Ft-M2 are not consistent with those previously reported. Johansson et al 2004 reported that the world-wide diversity of this locus (Nei's diversity index) is 0.58. Our estimated diversity (Simpson's Index of Diversity) for that locus was as high as 0.91 on Katama and 0.81 overall. The most parsimonious explanation is that homoplasy may occur at this locus. There are 22 distinct alleles, but similar alleles are found in the context of otherwise very diverse haplotypes. This particular locus on Martha's Vineyard appears to be mutating extremely quickly. It codes for a protein similar to *E. coli*'s anthranilate synthase component II but contains a frameshift rendering it inactive, and therefore the marker should not be under selective pressure. The current interpretation that the mutation rate is directly related to repeat copy number [36] may account for the large number of alleles we detected. In our study, the Ft-M2 locus has the greatest number of repeats (15–38) compared to all the other loci. The range of repeat copy number for all known *F. tularensis tularensis *strains, type AI, is 4–34 [21]. The diversity heretofore reported for this locus would appear to need revision when more strains with high copy numbers are included in subsequent analyses.

Bacterial population genetics and evolutionary theory provide testable hypotheses to address the basis for phenomena ranging from strain virulence to perpetuation. [38] To date, the population structure of *F. tularensis tularensis *would appear to be intractable, given the sporadic epizootic nature of outbreaks, other than at a scale based upon archival collections of isolates from across the United States. Our unique study site provides us with the first such analysis at a local scale that illuminates the mode of perpetuation of this bacterium in nature and which may give insights into the evolution of its capacity to cause severe disease.

## Conclusion

We demonstrate that tularemia natural foci can be genetically isolated even when located no more than 15 km apart in sites that have no physical barriers to biological interchange. The population structure at a site of stable transmission is that of a clonal complex, whereas an emergent focus derived from multiple founders. Stabilizing selection may act to homogenize population structure as a focus matures. It is likely that the agent of tularemia stably perpetuates in a metapopulation of isolated natural foci.

## Competing interests

The authors declare that they have no competing interests.

## Authors' contributions

All authors have reviewed and approved the final version of the paper. HKG designed the study, collected and processed the samples, conducted the data analysis and interpretation, and wrote the paper. BS assisted in processing the tick samples. SRT helped design the study, collect samples, and write the paper.

## References

[B1] Jellison W Tularemia in North America:1930–1974 1974 Missoula, MT: University of Montana

[B2] Farlow J Wagner DM Dukerich M Stanley M Chu M Kubota K Petersen J Keim P *Francisella tularensis *in the United States Emerg Infect Dis 2005 11 12 1835 1841 16485467 10.3201/eid1112.050728 PMC3367644

[B3] Keim P Johansson A Wagner DM Molecular epidemiology, evolution, and ecology of *Francisella* Annals Of The New York Academy Of Sciences 2007 1105 30 66 10.1196/annals.1409.011 17435120

[B4] Jellison WL Parker RR Rodents, rabbits and tularemia in North America – Some zoological and epidemiological considerations Amer J Trop Med 1945 25 4 349 362

[B5] Tularemia-United States 1990–2000 MMWR 2002 51 9 181 183 11900351

[B6] Bell JF The infection of ticks (*Dermacentor variabilis*) with *Pasteurella tularensis* J Infect Dis 1945 76 2 83 95

[B7] Parker RR Spencer RR Francis E Tularemia infection in ticks of the species *Dermacenter andersoni *Stiles in the Bitterroot Valley, Montana Pub Health Rep 1924 39 1057 1073

[B8] Hopla CE The multiplication of tularemia organisms in the lone-star tick Amer J Hyg 1955 61 3 371 380 14376386 10.1093/oxfordjournals.aje.a119761

[B9] Francis E Mayne B Experimental transmission of tularaemia by flies of the species *Chrysops discalis* Pub Health Rep 1921 36 1738 1746

[B10] Hopla CE Brankly CA, Cornelius C The ecology of tularemia Advances In Veterinary Science And Comparative Medicine 1974 18 New York, N.Y., U.S.A.; London, England: Academic Press 25 53 4419176

[B11] Matyas BI Nieder HS Telford SR Pneumonic tularemia on Martha's Vineyard – Clinical, epidemiologic, and ecological characteristics Francisella Tularensis: Biology, Pathogenicity, Epidemiology, And Biodefense 2007 1105 351 377 10.1196/annals.1409.013 17442781

[B12] Feldman KA Enscore RE Lathrop SL Matyas BT McGuill M Schriefer ME Stiles-Enos D Dennis DT Petersen LR Hayes EB An outbreak of primary pneumonic tularemia on Martha's Vineyard N Engl J Med 2001 345 22 1601 1606 10.1056/NEJMoa011374 11757506

[B13] Berrada ZL Goethert HK Telford SR Raccoons and skunks as sentinels for enzootic tularemia Emerg Infect Dis 2006 12 6 1019 1021 16707067 10.3201/eid1206.05879 PMC3373054

[B14] Goethert HK Shani I Telford SR Genotypic diversity of *Francisella tularensis *infecting *Dermacentor variabilis *ticks on Martha's Vineyard, Massachusetts J Clin Microbiol 2004 42 11 4968 4973 15528681 10.1128/JCM.42.11.4968-4973.2004 PMC525218

[B15] Johansson A Goransson I Larsson P Sjostedt A Extensive allelic variation among *Francisella tularensis *strains in a short-sequence tandem repeat region J Clin Microbiol 2001 39 9 3140 3146 11526142 10.1128/JCM.39.9.3140-3146.2001 PMC88310

[B16] Parker R Steinhaus E Kohls G Jellison W Contamination of Natural Waters and Mud with *Pasteurella tularensis *and Tularemia in Beavers and Muskrats in the Northwestern United States 1951 193 Washington, DC: US Government Printing Office 14869929

[B17] Goethert HK Telford SRI Nonrandom distribution of vector ticks (*Dermacentor variabilis*) infected by *Francisella tularensis* PLoS Pathog 2009 5 3 e1000319 19247435 10.1371/journal.ppat.1000319 PMC2642597

[B18] Davis S Klassovskiy N Ageyev V Suleimenov B Atshabar B Klassovskaya A Bennett M Leirs H Begon M Plague metapopulation dynamics in a natural reservoir: the burrow system as the unit of study Epidemiol Infect 2007 135 5 740 748 10.1017/S095026880600759X 17156497 PMC2870638

[B19] Gaff HD Gross LJ Modeling tick-borne disease: A metapopulation model Bull Math Biol 2007 69 1 265 288 10.1007/s11538-006-9125-5 17083005

[B20] Goethert HK Telford SR A new *Francisella *(*Beggiatiales: Francisellaceae*) inquiline within *Dermacentor variabilis *Say (*Acari: Ixodidae*) J Med Ent 2005 42 3 502 505 10.1603/0022-2585(2005)042[0502:ANFBFI]2.0.CO;2 15962806

[B21] Johansson A Farlow J Larsson P Dukerich M Chambers E Bystrom M Fox J Chu M Forsman M Sjostedt A Worldwide genetic relationships among *Francisella tularensis *isolates determined by multiple-locus variable-number tandem repeat analysis J Bacteriol 2004 186 17 5808 5818 15317786 10.1128/JB.186.17.5808-5818.2004 PMC516809

[B22] Hunter PR Gaston MA Numerical index of the discriminatory ability of typing systems: an application of Simpson's index of diversity J Clin Microbiol 1988 26 11 2465 2466 3069867 10.1128/jcm.26.11.2465-2466.1988 PMC266921

[B23] Feil E Li B Aanensen D Hanage W Spratt B eBURST: inferring patterns of evolutionary descent among clusters of related bacterial genotypes from multilocus sequence typing data J Bacteriol 2004 186 5 1518 1530 14973027 10.1128/JB.186.5.1518-1530.2004 PMC344416

[B24] Schouls LM Ende A van der Pol I van de Schot C Spanjaard L Vauterin P Wilderbeek D Witteveen S Increase in genetic diversity of *Haemophilus influenzae *serotype b (Hib) strains after introduction of hib vaccination in the Netherlands J Clin Microbiol 2005 43 6 2741 2749 15956392 10.1128/JCM.43.6.2741-2749.2005 PMC1151946

[B25] Slack A Symonds M Dohnt M Smythe L An improved multiple-locus variable number of tandem repeats analysis for *Leptospira interrogans *serovar Australis: a comparison with fluorescent amplified fragment length polymorphism analysis and its use to redefine the molecular epidemiology of this serovar in Queensland, Australia J Med Microbiol 2006 55 11 1549 1557 10.1099/jmm.0.46779-0 17030915

[B26] Agapow P-M Burt A Indices of multilocus linkage disequilibrium Mol Ecol Notes 2001 1 101 102 10.1046/j.1471-8278.2000.00014.x

[B27] Berdal BP Mehl R Meidell NK LorentzenStyr AM Scheel O Field investigations of tularemia in Norway FEMS Immunol Med Microbiol 1996 13 3 191 195 10.1111/j.1574-695X.1996.tb00235.x 8861027

[B28] Forsman M Henningson EW Larsson E Johansson T Sandstrom G *Francisella tularensis *does not manifest virulence in viable but non-culturable state FEMS Microbiol Ecol 2000 31 3 217 224 10.1111/j.1574-6941.2000.tb00686.x 10719202

[B29] Farlow J Smith KL Wong J Abrams M Lytle M Keim P *Francisella tularensis *strain typing using multiple-locus, variable-number tandem repeat analysis J Clin Microbiol 2001 39 9 3186 3192 11526148 10.1128/JCM.39.9.3186-3192.2001 PMC88316

[B30] Pavlovsky EN Natural Nidality of Transmissible Diseases 1966 Urbana: University of Illinois Press

[B31] Pollitzer R History and incidence of tularemia in the Soviet Union 1967 New York: Fordam University, Institute of Contemporary Russian Studies5633728

[B32] Sjostedt A Tularemia: History, epidemiology, pathogen physiology, and clinical manifestations Francisella Tularensis: Biology, Pathogenicity, Epidemiology, And Biodefense 2007 1105 Oxford: Blackwell Publishing 1 29 10.1196/annals.1409.009 17395726

[B33] Svensson K Back E Eliasson H Granberg M Guala D Karlsson L Larsson P Forsman M Johansson A *Francisella tularensis *genotypes correlate with fine scale geographical data during a natural outbreak of human tularemia 2007 Tularemia Workshop: 2007; Woods Hole, MA 2007

[B34] Tarnvik A Priebe HS Grunow R Tularaemia in Europe: An epidemiological overview Scand J Infect Dis 2004 36 5 350 355 10.1080/00365540410020442 15287379

[B35] Hopla CE Experimental studies on tick transmission of tularemia organisms Amer J Hyg 1953 58 1 101 118 13065276 10.1093/oxfordjournals.aje.a119585

[B36] Vogler AJ Keys C Nemoto Y Colman RE Jay Z Keim P Effect of repeat copy number on variable-number tandem repeat mutations in *Escherichia coli *O157: H7 J Bacteriol 2006 188 12 4253 4263 16740932 10.1128/JB.00001-06 PMC1482962

[B37] Vogler AJ Keys CE Allender C Bailey I Girard J Pearson T Smith KL Wagner DM Keim P Mutations, mutation rates, and evolution at the hypervariable VNTR loci of *Yersinia pestis* Mutat Res-Fund Mol M 2007 616 1–2 145 158 10.1016/j.mrfmmm.2006.11.007 17161849

[B38] Lipsitch M Microbiology – Bacterial population genetics and disease Science 2001 292 5514 59 60 10.1126/science.1060498 11294216

